# Supplementary material for: Ultrafast pseudospin quantum beats in multilayer WSe2 and MoSe2
Source: Nat Commun. 2022 Aug 25;13:4997. doi: 10.1038/s41467-022-32534-3 (PMC9411176; doi:10.1038/s41467-022-32534-3)
Supplement: Supplementary file 1 — Supplementary Information [file 41467_2022_32534_MOESM1_ESM.pdf]

Supplementary Information for:

Ultrafast pseudospin quantum beats in multilayer WSe_2 and $MoSe_2$

Simon Raiber,[1] Paulo E. Faria Junior,[2] Dennis Falter,[1] Simon Feldl,[1] Petter Marzena,[1]
Kenji Watanabe,[3] Takashi Taniguchi,[4] Jaroslav Fabian,[2] and Christian Schüller[1]

[1]*Institut für Experimentelle und Angewandte Physik,
Universität Regensburg, D-93040 Regensburg, Germany*

[2]*Institut für Theoretische Physik, Universität Regensburg, D-93040 Regensburg, Germany*

[3]*Research Center for Functional Materials,
National Institute for Materials Science, Tsukuba Ibaraki 305-0044, Japan*

[4]*International Center for Materials Nanoarchitectonics,
National Institute for Materials Science, Tsukuba Ibaraki 305-0044, Japan*

(Dated: July 1, 2022)

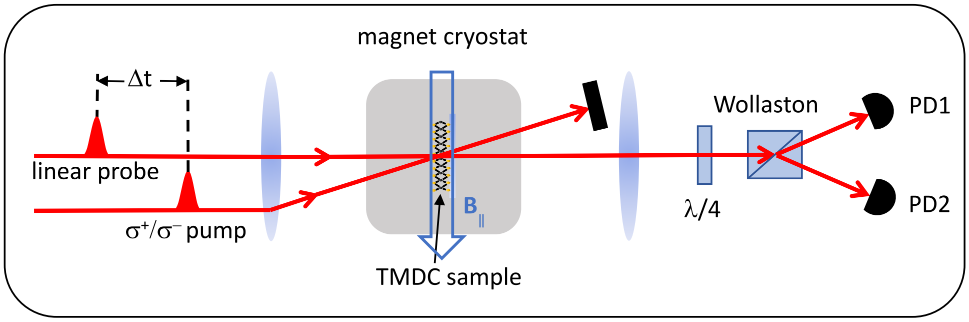

FIG. S1. Schematic picture of the experimental setup for time-resolved Faraday ellipticity experiments in external in-plane magnetic fields $\mathbf{B}_{\parallel}$.

Figure S1 shows the experimental setup, used for the time-resolved Faraday ellipticity (TRFE) experiments in external in-plane magnetic fields $B_{\parallel}$. For TRFE experiments, a mode-locked Ti:Sapphire laser is used, which produces laser pulses with a temporal length of about 80 fs at a repetition rate of 80 MHz. The laser beam is divided into two pulse trains by a beam splitter. The time delay, Δt, between pump and probe pulses is adjusted by a retroreflector, which is mounted on a linear stepper stage. Both beams are focused by a plano convex lens onto the sample surface, where they overlap. The laser spot diameter at the sample position is about 50 μm. The sample is mounted in an optical cryostat with superconducting magnet coils (split-coil cryostat) at a temperature of about $T = 5$ K, which is maintained by a constant flow of cold He gas. By measuring the laser pulse length before and after the magnet cryostat, we estimate the pulse length at the sample position to be about 130 fs. The pump pulses are circularly polarized and the laser wavelength is tuned to excitonic absorption lines to create a valley polarization in the sample. The temporal dynamics of the valley polarization is then measured by detecting the ellipticity of the linearly-polarized probe pulses after transmission of the sample. For measurement of the ellipticity, a combination of a Wollaston prism, quarter-wave plate and two balanced photo diodes is used. The pump beam is mechanically chopped at a frequency of about 1.6 kHz, and for detection of the photodiode difference signal, lockin technique is used.

In Fig. S2, a full TRFE dataset of the $MoSe_2$ multilayer sample with in-plane magnetic fields between 0 T and 9 T is shown. The pulsed laser was tuned in resonance with the A_{1s} exciton in the sample, and the pump pulses were σ^+ circularly polarized. The sharp spikes are measurement artifacts. Fig. S3 shows the same type of measurement but for the WSe_2 multilayer in resonance with the A_{2s} exciton. In Fig. S4, similar measurements, i.e., resonance with the A_{2s} exciton for different in-plane magnetic fields, are shown for the $MoSe_2$ multilayer.

For sample characterization, reflectance-contrast (RC) measurements of all samples are conducted in an optical microscope setup. The samples are mounted by an elastic organic glue on the cold finger of a He-flow cryostat and are kept in vacuum, while the sample holder is cooled down to nominally 5 K. The temperature at the sample position is estimated by the relative intensities of Ruby lines of the sapphire substrate. The substrate temperature is

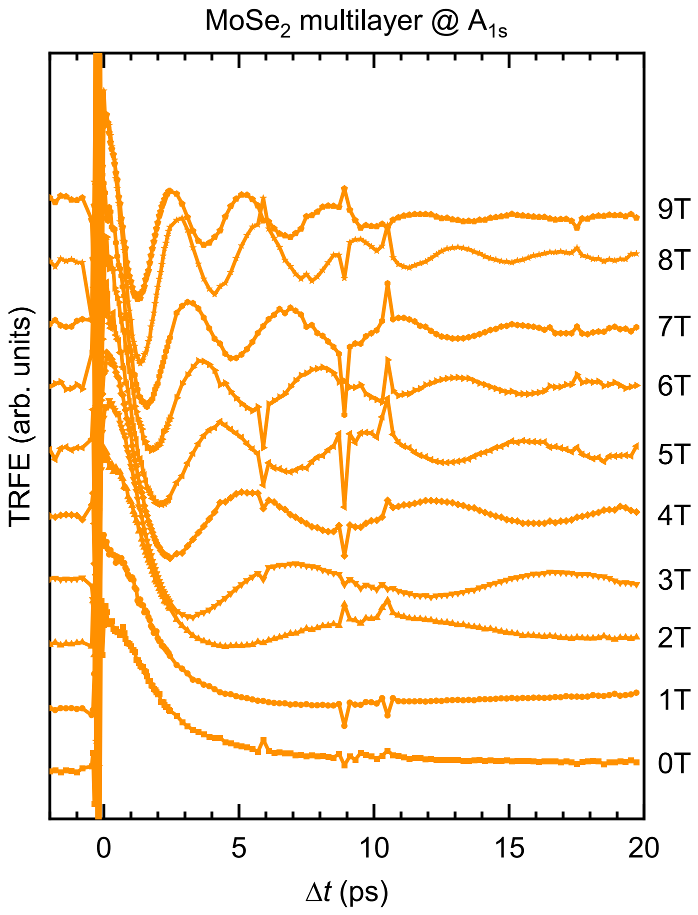

FIG. S2. TRFE traces of the $MoSe_2$ multilayer at different in-plane magnetic fields. The laser was tuned in resonance with the A_{1s} exciton, and the pump pulses were σ^+ polarized. The sharp spikes are measurement artifacts.

typically between about $T = 10$ K and 30 K. For the RC measurements, a white-light source is used, which is focused by a 60x microscope objective to a spot with diameter of about 10 μm. Reference spectra are recorded at positions next to the TMDC sample. Figure S5 shows RC spectra of the four samples, investigated in the main body of the manuscript. The RC spectra are evaluated as $\Delta R/(R_0 - R_{BG}) = (R - R_0)/(R_0 - R_{BG})$, where R is the reflectance of the sample, R_0 the reference spectrum, measured next to the sample, and R_{BG} the background dark spectrum.

For analysis of the RC spectra, fits using a transfer matrix model [1] are applied. The wavelength dependences of the refractive indices of hBN and sapphire are taken from Ref. [2].

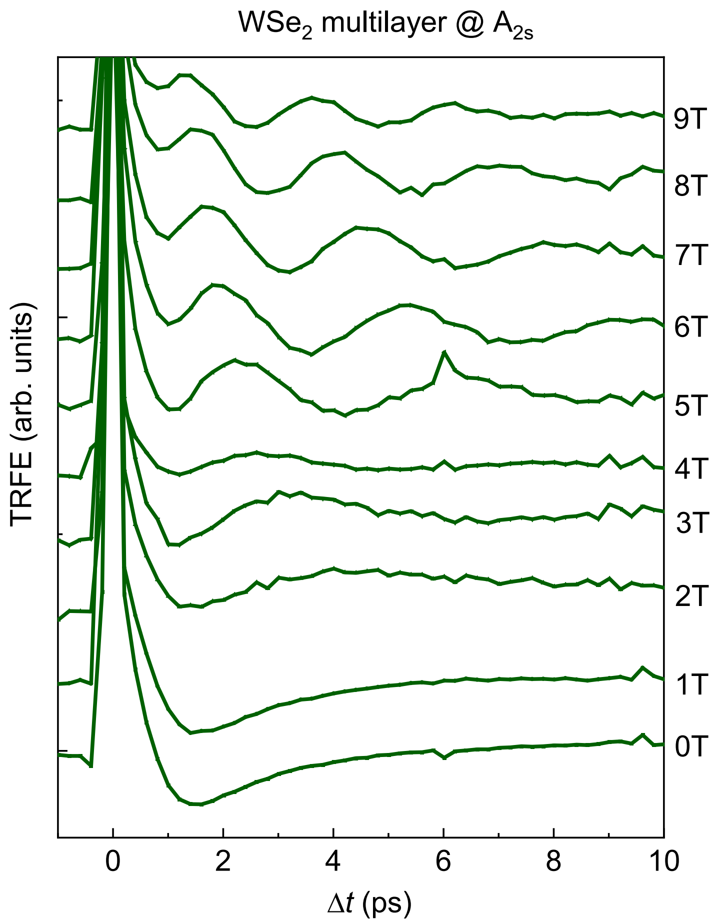

FIG. S3. TRFE traces of the WSe_2 multilayer at different in-plane magnetic fields. The laser was tuned in resonance with the A_{2s} exciton, and the pump pulses were σ^+ polarized.

For the TMDC layers, a parametrized model dielectric function ϵ_{TMDC} is used, where the excitonic transitions are modelled by complex Lorentz oscillators:

$$\epsilon_{\mathrm{TMDC}} = \epsilon_{\mathrm{BG}} + \sum_j \frac{f_j}{E^2(\lambda) - E_j^2 - iE\Gamma_j}$$

The index j is running over the relevant excitonic resonances, which, in our case, are up to three (see Fig. S5). The parameters of the Lorentzians are their oscillator strength f_j, the resonance energies E_j, and a phenomenological broadening Γ_j. ϵ_{BG} is a background dielectric constant. Figure S5 displays the measured RC spectra of the four samples, investigated in the main body of the manuscript. The red solid lines are fits with the transfer-matrix model. The numbers of used complex Lorentz oscillators are given in the figure. The positions of the oscillators are marked by vertical arrows and their energies are written next to the arrows.

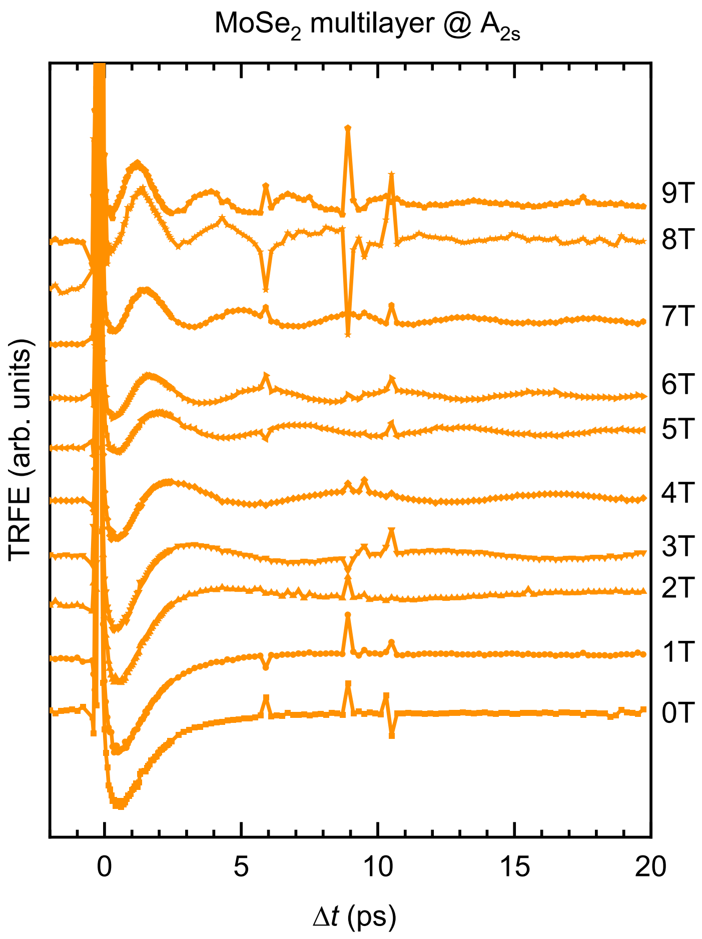

FIG. S4. TRFE traces of the $MoSe_2$ multilayer at different in-plane magnetic fields. The laser was tuned in resonance with the A_{2s} exciton, and the pump pulses were σ^+ polarized. The sharp spikes are measurement artifacts.

We have computed also the g factors for a WSe_2 bilayer, using the same computational details as discussed for monolayer and bulk with a vacuum region of 16 Å and an interlayer distance of 3.14 Å, the same as in the bulk. The corresponding values are displayed in table SI, together with the results for the monolayer and multilayer samples from the main text of the manuscript (table I of the manuscript). One can see that both, the values for $g_\parallel$ and $g_\perp$ of the bilayer are approximately in between the corresponding values for the monolayer and the multilayer samples. For the out-of-plane g factors, our calculated trend $|g_{\perp,\mathrm{monolayer}}| > |g_{\perp,\mathrm{bilayer}}| > |g_{\perp,\mathrm{bulk}}|$ is in agreement with layer dependent measurements from Arora et al. [3]. To test this experimentally, we have prepared a large-area WSe_2 bilayer, which is encapsulated in hBN. Figure S6a shows a microscope image of the sample.

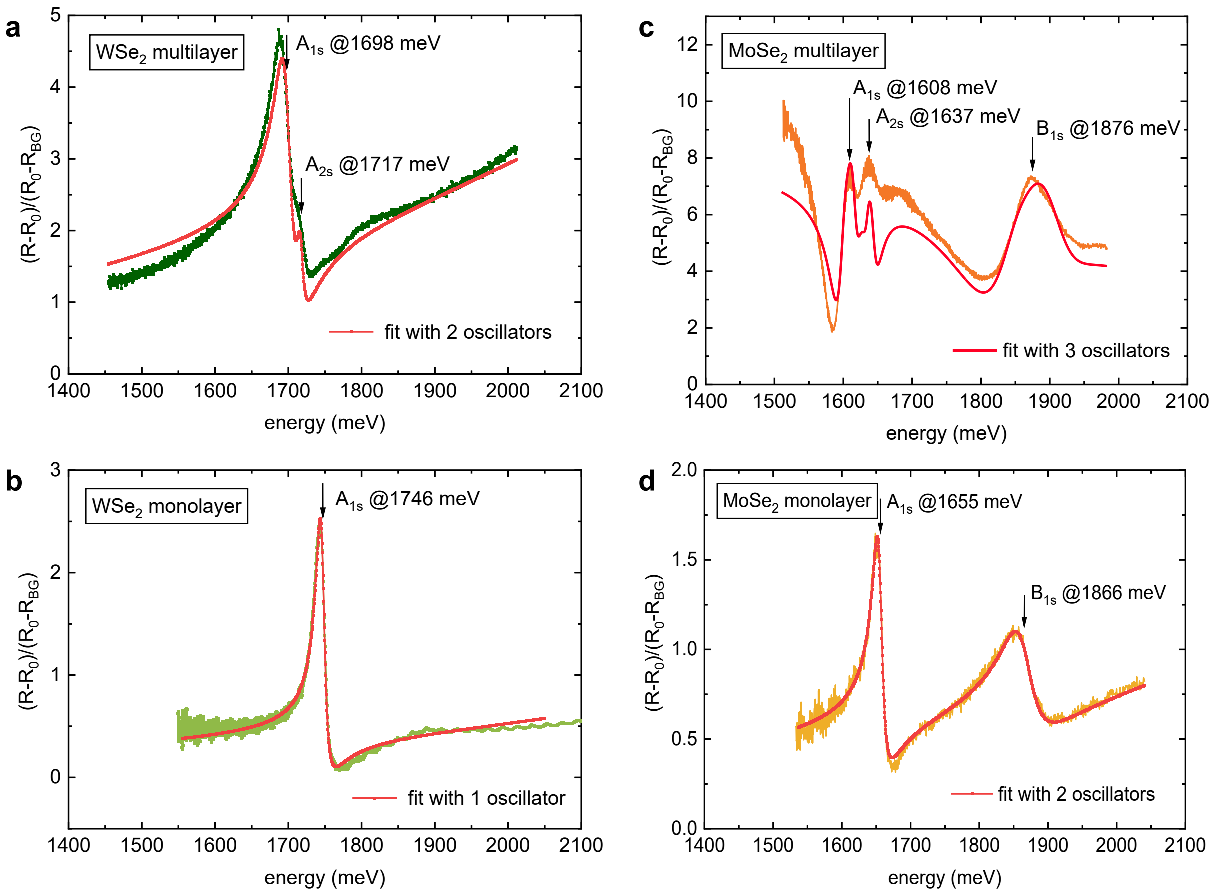

FIG. S5. Reflectance contrast spectra of the four samples, investigated in the manuscript. Results of fits with a transfer-matrix model, based on complex Lorentz oscillators for the excitonic transitions, are displayed by red solid lines. The numbers of used complex oscillators are given in the figure and their positions are marked by small vertical arrows.

Next to the bilayer is a thick (~ 100 layers) WSe_2 bulk part. The two dashed circles mark approximately the positions and sizes of the laser spots, used for TRFE experiments on the bilayer (blue circle) and the multilayer regions (orange circle). For the TRFE experiments, the laser wavelength was tuned to be in resonance with the A_{1s} excitons of the two materials. TRFE traces, measured on these spots for an in-plane magnetic field of $B_{\|} = 6$ T are shown in Figs. S6b and S6c for the bilayer and multilayer samples, respectively. While the temporal oscillations of the TRFE signal, known from the multilayer samples in the main part of the manuscript, can be nicely reproduced in the multilayer region, the signal of the bilayer does not show oscillations. As elaborated in the main part of the manuscript, we would expect

Material	S_z^{CB} L_z^{CB} S_z^{VB} L_z^{VB}	$g_\perp$	S_x^{CB} L_x^{CB} S_x^{VB} L_x^{VB}	$\lvert g_\parallel \rvert$
WSe_2 monolayer	0.98 2.97 1.00 5.00	−4.10	0.00 0.00 0.00 0.00	0.00
WSe_2 bilayer	0.97 2.93 1.00 4.76	−3.72	0.00 0.00 0.26 ±0.03	0.46 . . . 0.58
WSe_2 multilayer	0.97 2.98 1.00 4.40	−2.89	0.00 0.00 0.47 ±0.07	0.80 . . . 1.08

TABLE SI. Computed values of out-of-plane and in-plane spin-, S, and orbital, L, angular momenta for the conduction-band (CB) and valence-band (VB) states, which are relevant for the A excitons of WSe_2 materials. For the first-principles calculations, see the methods section. The corresponding theoretical g factors, $g_\perp$ and $|g_\parallel|$, for the A excitons are given.

oscillations also on the bilayer, though presumably with an approximately by a factor of two larger period, as obtained from the calculations above. We can only speculate about the absence of oscillations in the experiment on the bilayer. We would expect oscillations to occur for a symmetric bilayer sample, since there, the spin degeneracy is restored. The large-area encapsulated bilayer sample for sure has local inhomogeneities due to strain or varying dielectric environment because of better or worse local contact between the hBN and the bilayer. So, there may be a locally varying asymmetric potential, over which we average with our large ($\sim 50\mu$m) spot size. We believe that this may be the reason, hindering the appearance of pseudospin oscillations.

---

[1] S. J. Byrnes, Multilayer optical calculations, arXiv:1603.02720 (2016).

[2] https://refractiveindex.info

[3] A. Arora et al., 2D Materials **6**, 015010 (2019).

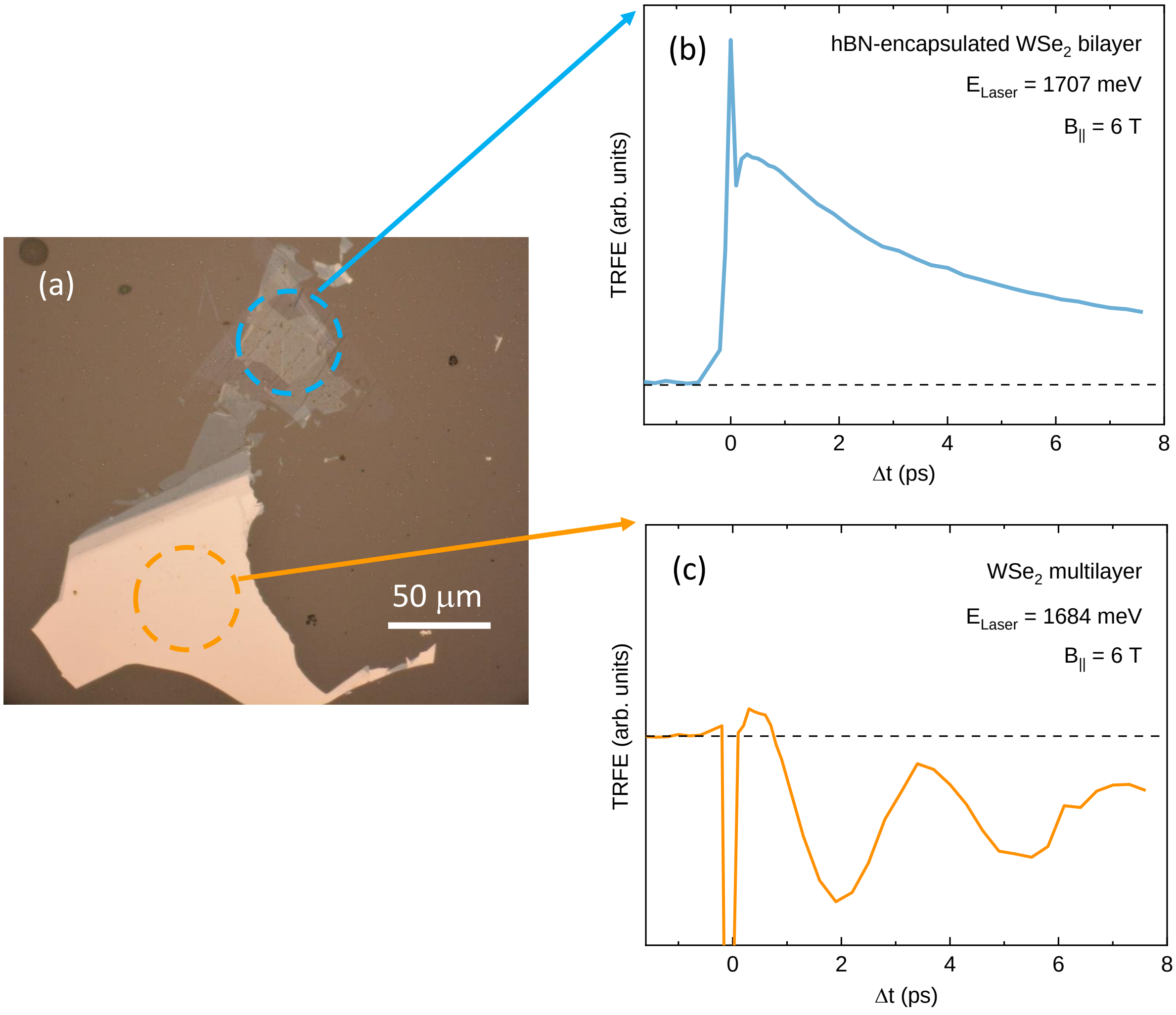

FIG. S6. (a) Microscope image of an hBN-encapsulated bilayer WSe_2 sample (upper, gray region) and a closeby bulk part (lower, bright region). The dashed circles mark positions of the laser spot for TRFE experiments in an in-plane magnetic field. (b) and (c) TRFE traces, measured on the sample spots, marked in (a), for an in-plane field of $B_{\parallel} = 6$ T. The laser energies are chosen to be in resonance with the A_{1s} exciton in the corresponding sample.
